# Effects of dietary leucine on growth, antioxidant capacity, immune response, and inflammation in juvenile yellow catfish *Pelteobagrus fulvidraco*

**DOI:** 10.3389/fphys.2023.1247410

**Published:** 2023-07-26

**Authors:** Dexiang Feng, Yangping Yu, Kaifang Liu, Yi Su, Tianyu Fan, Xusheng Guo, Ming Li

**Affiliations:** ^1^ School of Fisheries, Xinyang Agriculture and Forestry University, Xinyang, China; ^2^ School of Marine Sciences, Ningbo University, Ningbo, China

**Keywords:** leucine, growth, antioxidant enzyme, immune response, *Pelteobagrus fulvidraco*

## Abstract

The experiment was conducted to investigate the effects of dietary leucine on growth, antioxidant capacity, immune response, and inflammation in juvenile yellow catfish. Five diets were formulated to contain five dietary leucine levels: 12.00 (control), 19.00, 26.00, 33.00, and 40.00 g kg^−1^. Each diet was randomly assigned to triplicate groups of 30 juvenile fish (5.02 ± 0.15 g) twice daily to apparent satiation for 56 days. Weight gain rate, specific growth rate, and activities of liver superoxide dismutase, glutathione peroxidase, and serum lysozyme, as well as immunoglobulin M content, significantly increased with increase in dietary leucine levels up to 26.00 g kg^−1^, but those values decreased significantly with a further increase in dietary leucine. On the contrary, the lowest malondialdehyde content was found in 26.00 and 33.00 g kg^−1^ leucine groups. The expression levels of *IGF 1* and *MYF 5* genes in muscle were significantly upregulated with increase in dietary leucine levels up to 26.00 g kg^−1^, but the expression of *MSTN* level showed the opposite trend. The lowest expression levels of *IL 8* and *TNFɑ* genes in the liver were found in 26.00 g kg^−1^ leucine groups. The quadratic regression analysis on weight gain, specific growth rate, and feed conversion ratio against dietary leucine levels indicated that the optimal dietary leucine requirement was estimated to be 26.84–27.00 g kg^−1^of the dry diet.

## 1 Introduction

Leucine (Leu) is a branched-chain amino acid, which is one of the amino acids required in fish nutrition (Yin et al., 2010; Duan et al., 2016). The results of earlier studies have shown that the addition of appropriate leucine to dietary additives can encourage the growth of fish. For example, dietary leucine contents of 14.00–15.60 g kg^−1^ can improve weight gain of blunt snout bream *Megalobrama amblycephala* (Liang et al., 2017). Dietary leucine contents of 23.50–23.90 g kg^−1^ can increase the weight gain rate and specific growth rate of black carp *Mylopharyngodon piceus* (Wu et al., 2017). Leucine is an activator of growth-related genes, including *MYOD*, *MYF5*, and *MYOG* (Han et al., 2010). The *MYOD* and *MYF5* genes are involved in the formation of myocytes, and the *MYOG* sustainably actuated the differentiation of myocytes and promoted the formation and enlargement of myotubes (Valente et al., 2013). It should be noted that the *MSTN* gene was a negative regulator, playing a role in the proliferation and differentiation of myocytes, while leucine can counteract the negative regulatory effect of *MSTN* and guarantee the normal proliferation of muscle fibers (Mobley et al., 2014). In mice, leucine can upregulate *MYOD*, *MYF5*, and *MYOG* gene expression in the muscle, thus accelerating the proliferation of muscle cells and regeneration of skeletal muscle (Perry et al., 2014). However, similar studies have been less reported in fish, and only one study reported that dietary leucine content of 25.20 g kg^−1^ can upregulate *IGF 1* expression in the muscle of largemouth bass *Micropterus salmoides* (Yang et al., 2022). The growth characteristics of fish are different from those of mammals, so the growth-promoting mechanism of leucine is worth further study.

A previous study found that dietary leucine deficiency would induce superabundant production of activated oxygen, which is toxic to organisms (Tan et al., 2016). Generally, fish have an effective antioxidant system, including superoxide dismutase, catalase, and glutathione peroxidase, which can remove reactive oxygen species to prevent oxidative damage (Madeira et al., 2013). Earlier studies have reported that optimal supplementation of dietary leucine can increase liver total antioxidant capacity, superoxide dismutase, catalase, and glutathione peroxidase activities and reduce malondialdehyde content in blunt snout bream (14.00–15.60 g kg^−1^) (Deng et al., 2014; Liang et al., 2017) and grass carp *Ctenopharyngodon idella* (13.3 g kg^−1^) (Deng et al., 2014). In addition, leucine also affects immune response and inflammatory response in fish. For example, dietary leucine supplementation improved serum lysozyme, acid phosphatase, and alkaline phosphatase activities and complemented immunoglobulin M contents in grass carp (12.90 g kg^−1^) and black carp (23.50–23.90 g kg^−1^) (Jiang et al., 2015; Wu et al., 2017). Dietary leucine supplementation can downregulate the mRNA expression level of *TNF α*, *IL 1*, and *IL 8* in golden pompano *Trachinotus ovatus* (29.30–32.90 g kg^−1^) (Tan et al., 2016), grass carp (23.50–23.90 g kg^−1^) (Jiang et al., 2015), rohu; *Labeo rohita* (4.60 g kg^−1^) (Giri et al., 2015), and blunt snout bream (14.00–15.60 g kg^−1^) (Liang et al., 2017). Different fish have different optimal leucine requirements, so it is crucial to understand the leucine requirements of typical fish species.

Yellow catfish is popular for its economic value in southern China (Qiang et al., 2019). However, the optimum dietary supplemental level of leucine in yellow catfish has not been reported so far. The objective of the study was to evaluate the impact of leucine levels in feed on growth performance, body composition, antioxidant status, immune response, and related gene expression levels of yellow catfish and to provide an important reference for the optimal nutritional requirements of this species.

## 2 Materials and methods

### 2.1 Experimental diets

Five experimental diets were designed and supplemented with five different levels of leucine: 12.00 (control), 19.00, 26.00, 33.00, and 40.00 g kg^−1^. The protein sources were provided by fish meal, rice concentrate protein, and corn gluten meal, and the lipid sources were provided by fish oil and soybean oil (Table 1). The solid material is ground and passed through a 60-mesh (0.25 mm) screen before application. All raw materials were carefully weighed and mixed and then extruded to sinking pellets (2 mm) with a feed mill (F-26II, Science and Technology Industrial General Factory of South China University of Technology, China). The experimental diets were air-dried and stored at −20°C.

**TABLE 1:** Composition and nutrient content of experimental diets (g kg^−1^ dry matter).

	Leucine level				
	12.00	19.00	26.00	33.00	40.00
Ingredients					
Fish meal	180.00	180.00	180.00	180.00	180.00
Rice protein concentrate	360.00	360.00	360.00	360.00	360.00
Corn gluten meal	50.00	50.00	50.00	50.00	50.00
Wheat meal	200.00	200.00	200.00	200.00	200.00
Fish oil	10.00	10.00	10.00	10.00	10.00
Soybean oil	10.00	10.00	10.00	10.00	10.00
Choline chloride	5.00	5.00	5.00	5.00	5.00
Vitamin mixture^1^	5.00	5.00	5.00	5.00	5.00
Mineral mixture^2^	5.00	5.00	5.00	5.00	5.00
Sodium carboxymethyl cellulose	20.00	20.00	20.00	20.00	20.00
Cellulose	124.50	124.50	124.50	124.50	124.50
Leucine	0.00	7.50	15.00	22.50	30.00
Alanine	30.00	22.50	15.00	7.50	0.00
Ethoxy quinoline	0.50	0.50	0.50	0.50	0.50
Proximate composition					
Protein (*N* × 6.25)	38.48	38.56	38.33	38.76	38.61
Lipid	7.49	7.50	7.47	7.52	7.58
Leucine	11.05	20.01	25.88	32.78	39.81

^a^ Vitamin premix (IU or g kg^−1^ premix): retinyl acetate, 3,500,000 IU; cholecalciferol, 450,000 IU; ɑ-tocopherol, 7500 IU; thiamine, 10 g; riboflavin, 6 g; pyridoxine hydrochloride, 12 g; nicotinic acid, 40 g; d-calcium pantothenate, 15 g; biotin, 0.25 g; folic acid, 0.4 g; inositol, 200 g; cyanocobalamin 0.02 g; menadione, 4 g.

^b^ Mineral premix (g kg^−1^premix): FeC_6_H_5_O_7_, 5.21; ZnSO_4_•7H_2_O, 8.76; MnSO_4_•H_2_O, 4.14; CuSO_4_•5H_2_O, 6.61; MgSO_4_•7H_2_O, 238.97; KH_2_PO_4_, 233.2; NaH_2_PO_4_, 137.03; C_6_H_10_CaO_6_•5H_2_0, 34.09; CoCl_2_•6H_2_O, 1.36.

### 2.2 Experimental animals

Juvenile yellow catfish were collected from Xinyang, Henan, and adaptively incubated in cylindrical buckets (diameter of 1.5 m) for 2 weeks. The experiment was conducted in 15 square plastic buckets (0.8 × 0.8 × 0.6 m) with three replicates per treatment group, and each bucket was filled with 250 L of water. Thirty fish (5.02 ± 0.15 g) were randomly assigned to each bucket. Each bucket is filled with cylindrical plastic tubes 10 cm in diameter, which serve as a habitat for yellow catfish. Air stones and pumps provide sufficient oxygen. Experimental diets were fed to apparent satiety at 7:30 a.m. and 16:30 p.m. for 56 days, and one-third of new water was replaced every 7 days. The water temperature was maintained at 28.00°C ± 2.00°C, dissolved oxygen >8.00 ± 0.20 mg L^−1^, ammonia <0.01 mg L^−1^, and nitrite <0.05 mg L^−1^.

### 2.3 Sample collection

Feeding was stopped for 24 h before sampling; then fish were anesthetized with MS-222; and then the weight of each fish, the number of fish per barrel, and the length and height of each fish were measured. To profile the proximate composition of the whole body, five fish were randomly picked from each pail and were stored at −20°C. Blood plasma (three fish per bucket) is collected from the end of the tail vein using a syringe moistened with sodium heparin. After centrifugation at 3,500 rpm min^−1^, serum was taken and stored at −20°C for the determination of immune indexes. After being excised, fresh livers were stored at −80°C for qPCR analysis and at −20°C for the antioxidant enzyme activity assay. Then, the muscle also was removed and preserved at −80°C for qPCR analysis.

### 2.4 Biochemical analysis

All experimental diets and experimental samples were analyzed for approximate composition using standard methods (Official Society of Analytical Chemists) (AOAC, 1990).

The liver tissue block was rinsed with phosphoric acid buffer (PBS), and 0.1 g of liver tissue was weighed and added into PBS according to the ratio of weight/volume = 1/9. The liver tissue was mixed with a crushing homogenizer (IKA T10 BASIC), and then 10% of the liver tissue homogenate was prepared in a sterile and ice-cold water bath environment. The tissue was centrifuged at 2,800 g, 4°C for 10 min, and the upper supernatant was collected. Total antioxidant capacity, superoxide dismutase, catalase, glutathione peroxidase activities, and malondialdehyde content were analyzed using commercial reagent kits (Nanjing Jianchen Bioengineering Institute, China).

Serum lysozyme, acid phosphatase, alkaline phosphatase activities, 50% hemolytic complement, and immunoglobulin M were detected by commercial reagent kits (Nanjing Jiancheng Bioengineering Institute, Nanjing, China). The enzymatic reaction is carried out at 28°C.

### 2.5 Real-time PCR analysis

Extraction of total RNA from the liver and muscle was performed with an RNAiso reagent kit (Vazyme Biotech Company, China). RNA quality was determined by a microultraviolet spectrophotometer (ND 2000; Thermo Fisher Scientific Incorporated, China) at a 260/280 ratio, and then detection of RNA integrity by two percent agarose gel electrophoresis was carried out. Reverse transcription of the first cDNA was performed using the Prime Script™ RT kit (Takara Bio Company, China). The primer systems used for real-time PCR are shown in Table 2. The real-time PCR was carried out in a Roche LightCycler^®^ 480 II real-time PCR machine (Roche Limited, Switzerland) using SYBR Premix Ex Taq (Takara Bio Company, China), containing 10 μL Ultra SYBR Mixture, 0.4 μL of cDNA, 8.8 μL of dH_2_O, 0.4 μL of forward primer, and 0.4 μL of reverse primer. Reaction conditions for qPCR are as follows: 95°C for 45 s, followed by 35 cycles at 95°C for 10 s and 60°C for 20 s, with a melting curve of 95°C for 10 s and 60°C for 1 min, and *β-actin* and *GAPDH* as house-keeping genes. The relative expression of genes was calculated using the 2^−ΔΔCT^ assay (Livak and Schmittgen, 2001).

**TABLE 2:** Primers used for real-time PCR analysis from yellow catfish.

Target gene	Primer sequence (5′-3′)	Size (bp)
Growth-related genes		
*IGF 1*	F: GCA​CAA​CCG​TGG​CAT​TGT​AG	135
	R: GAC​GTG​TCT​GTG​TGC​CGT​T	
*MYF 5*	F: GGC​TAG​AGA​AGG​TGA​ACC​AC	290
	R: CGC​ACT​CTG​ACC​TTC​GTA​AC	
*MYOD*	F: TAT​TCC​GTT​CCC​CAT​CCC​CT	208
	R: TTT​ACA​CGC​CCA​CAG​GAG​AC	
*MYOG*	F: ACC​CGT​ACT​TTT​TCC​CCG​AA	129
	R: CAT​CCC​CAC​ATA​GCC​CTA​CC	
*MSTN*	F: GCG​CAC​CAA​GAG​AGA​ATC​AG	125
	R: AGC​GTT​TCG​GGG​CAA​TAA​TC	
Inflammatory response genes		
*IL 1*	F: GGC​TGG​TTT​GCT​GAT​GTG​TC	101
	R: CTC​GCT​GAA​CAC​CTT​CGA​GT	
*IL 8*	F: CAC​TCA​CCA​AGC​CAG​CAA​TG	228
	R: AGA​CAA​CCC​AAG​ACT​TCA​CC	
*TNFα*	F: ATC​AGG​TGA​ACG​CTG​ATG​CT	98
	R: GTG​TTG​AGG​GAA​GGG​GTC​TG	
House-keeping genes		
*β-Actin*	F: TTCGCTGGAGATGATGCT	136
	R: CGTGCTCAATGGGGTACT	
*GAPDH*	F: TCT​GGG​GTA​CAC​AGA​ACA​CC	165
	R: ACT​AGG​TCA​CAG​ACA​CGG​TT	

### 2.6 Statistical analyses

The specimen data are represented as a mean ± standard error (mean ± SD). SPSS 24.0.0 software (Chicago, United States) from Windows was used for one-way ANOVA, when the difference reached the level of significance (*p* < 0.05), Duncan’s method was used for multiple comparison. The correlation analysis was performed using Pearson’s analysis. The R package is utilized to conduct correlation analysis and generate heat maps, and bar charts were plotted by GraphPad Prism 8 software (San Diego, United States).

## 3 Results

### 3.1 Growth performance

**FIGURE 1:**
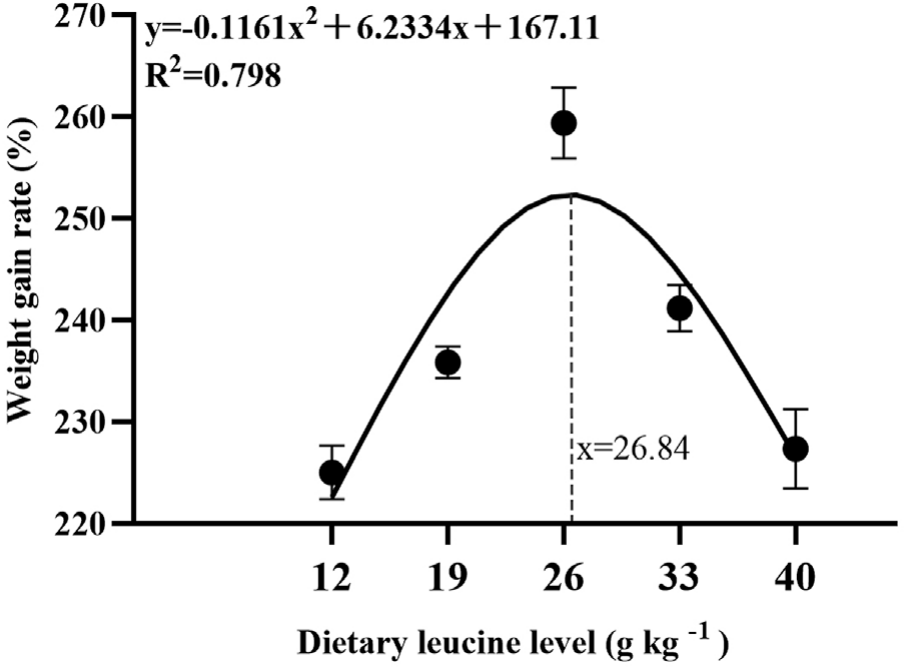
Quadratic regression analysis of weight gain rate (WGR) in juvenile yellow catfish fed diets supplemented with various levels of leucine for 56 days.

**FIGURE 2:**
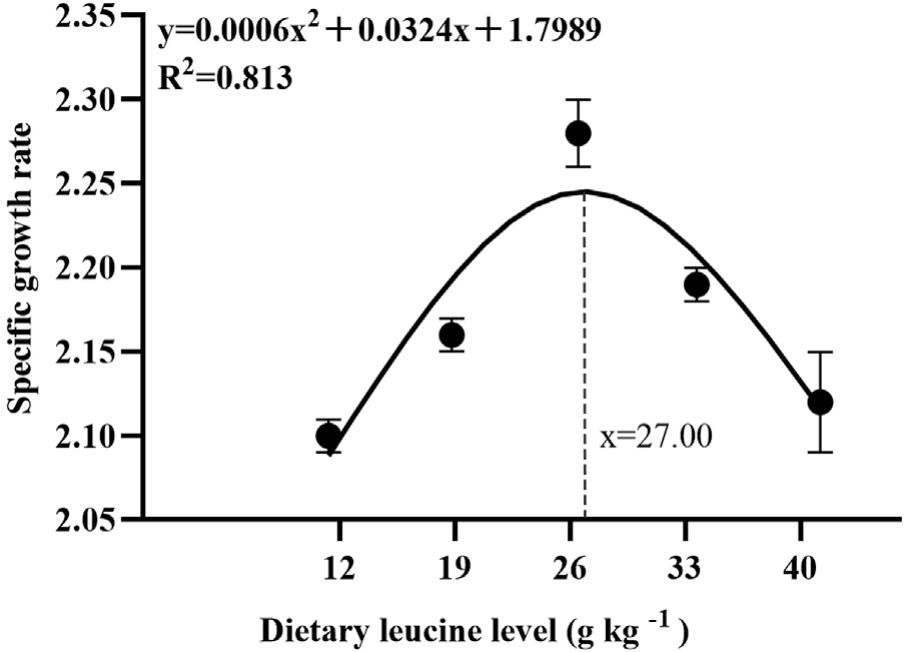
Quadratic regression analysis of the specific growth rate (SGR) in juvenile yellow catfish fed diets supplemented with various levels of leucine for 56 days.

**FIGURE 3:**
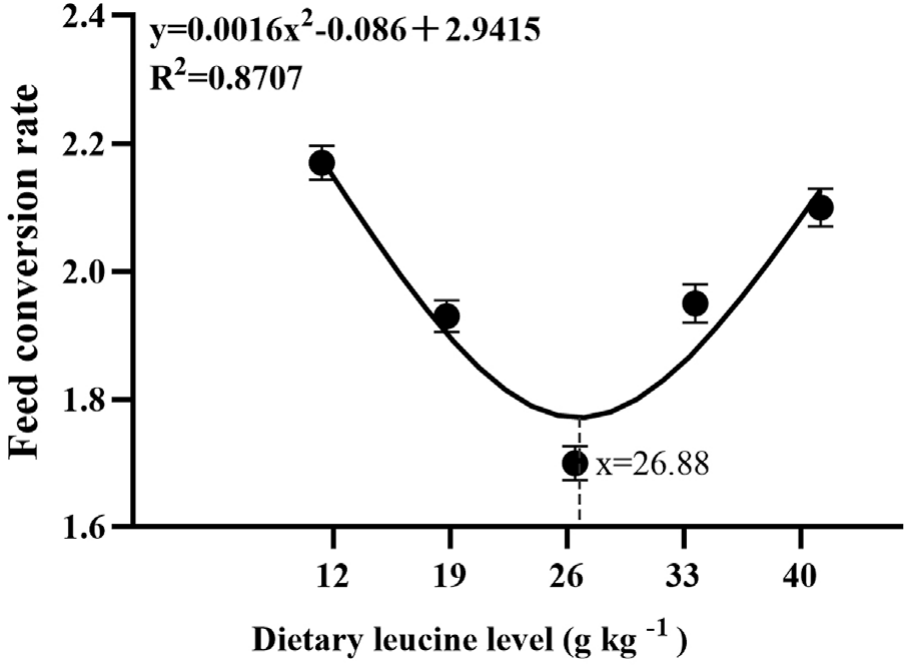
Quadratic regression analysis of the feed conversion ratio (FCR) in juvenile yellow catfish fed diets supplemented with various levels of leucine for 56 days.

As shown in Table 3, the weight gain rate and specific growth rate significantly increased with increase in dietary leucine levels up to 26.00 g kg^−1^, but that the value declined significantly with further increments in dietary leucine level (*p* < 0.05). The feed conversion ratio significantly decreased with increase in dietary leucine levels to 26.00 g kg^−1^ (*p* < 0.05). The maximum values of the condition factor, hepatosomatic index, and viscerosomatic index were observed in the 26.00 g kg^−1^ group (*p* < 0.05). Leucine levels did not affect the survival rate, which was above 95% (*p* > 0.05). Quadratic regression analysis between the weight gain rate contact dietary leucine levels showed of 26.84 g kg^−1^ leucine of the diet was required for optimal growth of yellow catfish (y = −0.1161x^2^ + 6.2334x + 167.11, *R* ^2^ = 0.798) (Figure 1). Quadratic regression analysis between the specific growth rate against dietary leucine levels showed that diet containing 27.00 g kg^−1^ leucine was required for optimal growth of yellow catfish (y = −0.0006 x^2^ + 0.0324 x + 1.7989, *R* ^2^ = 0.813) (Figure 2). Quadratic regression analysis between the feed conversion ratio against dietary leucine levels showed that diet containing 26.88 g kg^−1^ leucine was required for the optimal growth of yellow catfish (y = 0.0016x^2^−0.0864x + 2.9415, *R* ^2^ = 0.8707) (Figure 3).

**TABLE 3:** Growth performance of juvenile yellow catfish fed diets supplemented with various levels of leucine for 56 days.

	Leucine level (g kg^−1^)				
	12.00	19.00	26.00	33.00	40.00
FW (g)	16.27 ± 0.04^c^	16.80 ± 0.03^b^	17.99 ± 0.09^a^	17.04 ± 0.19^b^	16.34 ± 0.21^c^
WGR (%)	225.04 ± 0.64^c^	235.85 ± 1.55^b^	259.40 ± 3.49^a^	241.18 ± 2.30^b^	227.34 ± 6.87^c^
SGR	1.88 ± 0.01^c^	1.97 ± 0.01^b^	2.16 ± 0.02^a^	2.01 ± 0.03^b^	1.89 ± 0.04^c^
FCR	2.17 ± 0.02^a^	1.93 ± 0.02^c^	1.70 ± 0.01^d^	1.95 ± 0.03^c^	2.10 ± 0.03^b^
CF	1.37 ± 0.04^c^	1.43 ± 0.01^b^	1.51 ± 0.03^a^	1.45 ± 0.03^b^	1.42 ± 0.02^bc^
HSI (%)	1.13 ± 0.04^b^	1.12 ± 0.04^b^	1.22 ± 0.03^a^	1.16 ± 0.06^ab^	1.14 ± 0.04^b^
VSI (%)	8.05 ± 0.05^bc^	8.12 ± 0.09^b^	8.30 ± 0.05^a^	8.18 ± 0.06^b^	8.04 ± 0.02^c^
SR (%)	95.83 ± 1.44	95.83 ± 1.44	95.00 ± 2.50	96.67 ± 1.44	96.67 ± 1.56

Data are expressed as mean value ±SD (*n* = 3). Means in the same row with different superscripts (a, b, c) are significantly different (*p* < 0.05). FW, final weight; weight gain rate (WGR, %) = (final weight−initial weight)/initial weight × 100; specific growth rate (SGR) = [(Ln final weight−Ln initial weight)/days] × 100; feed conversion ratio (FCR) = dry diet feed/wet weight gain; condition factor (CF) = body weight/body length^3^ ×100; hepatosomatic index (HSI, %) = liver weight/body weight × 100; viscerosomatic index (VSI, %) = viscera weight/body weight × 100; Survival rate (SR, %) = number of surviving fish/numbers of total fish × 100.

As shown in Table 4, the moisture content of 26.00 and 33.00 g kg^−1^ groups was significantly lower than that of the other groups (*p* < 0.05). The crude protein and crude lipid contents in 26.00 and 33.00 g kg^−1^ groups were significantly higher than those in other groups (*p* < 0.05). There was no significant difference in ash content among all groups (*p* > 0.05).

**TABLE 4:** Whole-body composition of juvenile yellow catfish fed diets supplemented with various levels of leucine for 56 days.

	Leucine level (g kg^−1^)				
	12.00	19.00	26.00	33.00	40.00
Moisture (%)	72.52 ± 1.17^a^	70.37 ± 0.27^b^	68.61 ± 1.37^c^	69.87 ± 0.17^bc^	72.70 ± 0.51^a^
Protein (% dry matter)	16.14 ± 0.18^c^	16.39 ± 0.18^bc^	16.84 ± 0.14^a^	16.79 ± 0.11^a^	16.47 ± 0.20^b^
Lipid (% dry matter)	8.35 ± 0.11^d^	8.64 ± 0.10^c^	9.09 ± 0.15^a^	8.89 ± 0.13^ab^	8.73 ± 0.07^bc^
Ash (% dry matter)	3.13 ± 0.01	3.17 ± 0.09	3.17 ± 0.13	3.15 ± 0.01	3.08 ± 0.03

Data are expressed as mean value ±SD (*n* = 3). Means in the same row with different superscripts (a, b, c) are significantly different (*p* < 0.05).

### 3.2 Liver antioxidant enzyme activity

As shown in Table 5, liver superoxide dismutase and glutathione peroxidase activities significantly increased as dietary leucine levels increased up to 26.00 g kg^−1^, but those values decreased significantly with a further increase in dietary leucine (*p* < 0.05). The total antioxidant capacity and catalase activity in 26.00 and 33.00 g kg^−1^ groups were significantly higher than those in other groups (*p* < 0.05). On the contrary, the lowest value of malondialdehyde was found in 26.00 and 33.00 g kg^−1^ groups (*p* < 0.05).

**TABLE 5:** Liver antioxidant enzyme activity of juvenile yellow catfish fed diets supplemented with various levels of leucine for 56 days.

	Leucine level (g kg^−1^)				
	12.00	19.00	26.00	33.00	40.00
T-AOC (U mg^−1^ protein)	1.93 ± 0.13^c^	2.24 ± 0.12^b^	2.51 ± 0.20^a^	2.41 ± 0.09^ab^	1.98 ± 0.05^c^
SOD (U mg^−1^ protein)	91.07 ± 1.35^d^	98.29 ± 0.54^bc^	109.59 ± 1.28^a^	100.61 ± 1.34^b^	96.23 ± 2.82^c^
CAT (U mg^−1^ protein)	3.12 ± 0.22^c^	3.45 ± 0.09^b^	4.06 ± 0.23^a^	3.85 ± 0.13^a^	3.53 ± 0.10^b^
GPx (U mg^−1^ protein)	55.13 ± 2.09^c^	63.66 ± 1.54^b^	76.06 ± 3.02^a^	68.04 ± 3.77^b^	64.56 ± 1.53^b^
MDA (nmol mg^−1^ protein)	5.49 ± 0.09^a^	4.82 ± 0.11^b^	3.75 ± 0.12^d^	3.84 ± 0.06^d^	4.16 ± 0.05^c^

Data are expressed as mean value ±SD (*n* = 3). Means in the same row with different superscripts (a, b, c) are significantly different (*p* < 0.05). T-AOC, total antioxidant capacity; SOD, superoxide dismutase; CAT, catalase; GPx, glutathione peroxidase; MDA, malondialdehyde.

### 3.3 Serum immune response

As shown in Table 6, serum lysozyme, acid phosphatase and alkaline phosphatase activities, 50% hemolytic complement, and immunoglobulin contents significantly increased with increase in dietary leucine levels up to 26.00 g kg^−1^, but those values decreased significantly with a further increase in dietary leucine (*p* < 0.05).

**TABLE 6:** Serum immune response of juvenile yellow catfish fed diets supplemented with various levels of leucine for 56 days.

	Leucine level (g kg^−1^)				
	12.00	19.00	26.00	33.00	40.00
LYZ (U mL^−1^)	72.55 ± 1.59^d^	86.75 ± 5.78^bc^	97.49 ± 1.31^a^	88.14 ± 1.67^b^	80.17 ± 5.05^c^
ACP (U mL^−1^)	35.26 ± 0.58^c^	40.38 ± 3.35^b^	46.58 ± 3.26^a^	43.18 ± 1.00^ab^	42.77 ± 2.12^ab^
AKP (U mL^−1^)	77.94 ± 4.32^d^	92.33 ± 2.08^b^	98.72 ± 1.38^a^	95.52 ± 4.85^ab^	85.53 ± 1.38^c^
CH50 (mg mL^−1^)	17.09 ± 1.77^b^	20.65 ± 1.79^ab^	21.85 ± 2.74^a^	18.86 ± 1.78^ab^	20.05 ± 1.03^ab^
IgM (mg mL^−1^)	43.49 ± 3.96^c^	52.99 ± 2.59^b^	60.77 ± 2.59^a^	51.26 ± 3.96^b^	50.40 ± 4.49^b^

Data are expressed as mean value ±SD (*n* = 3). Means in the same row with different superscripts (a, b, c) are significantly different (*p* < 0.05). LYZ, lysozyme; ACP, acid phosphatase; AKP, alkaline phosphatase; CH50, 50% hemolytic complement; IgM, immunoglobulin M.

### 3.4 Muscle growth gene expression

**FIGURE 4:**
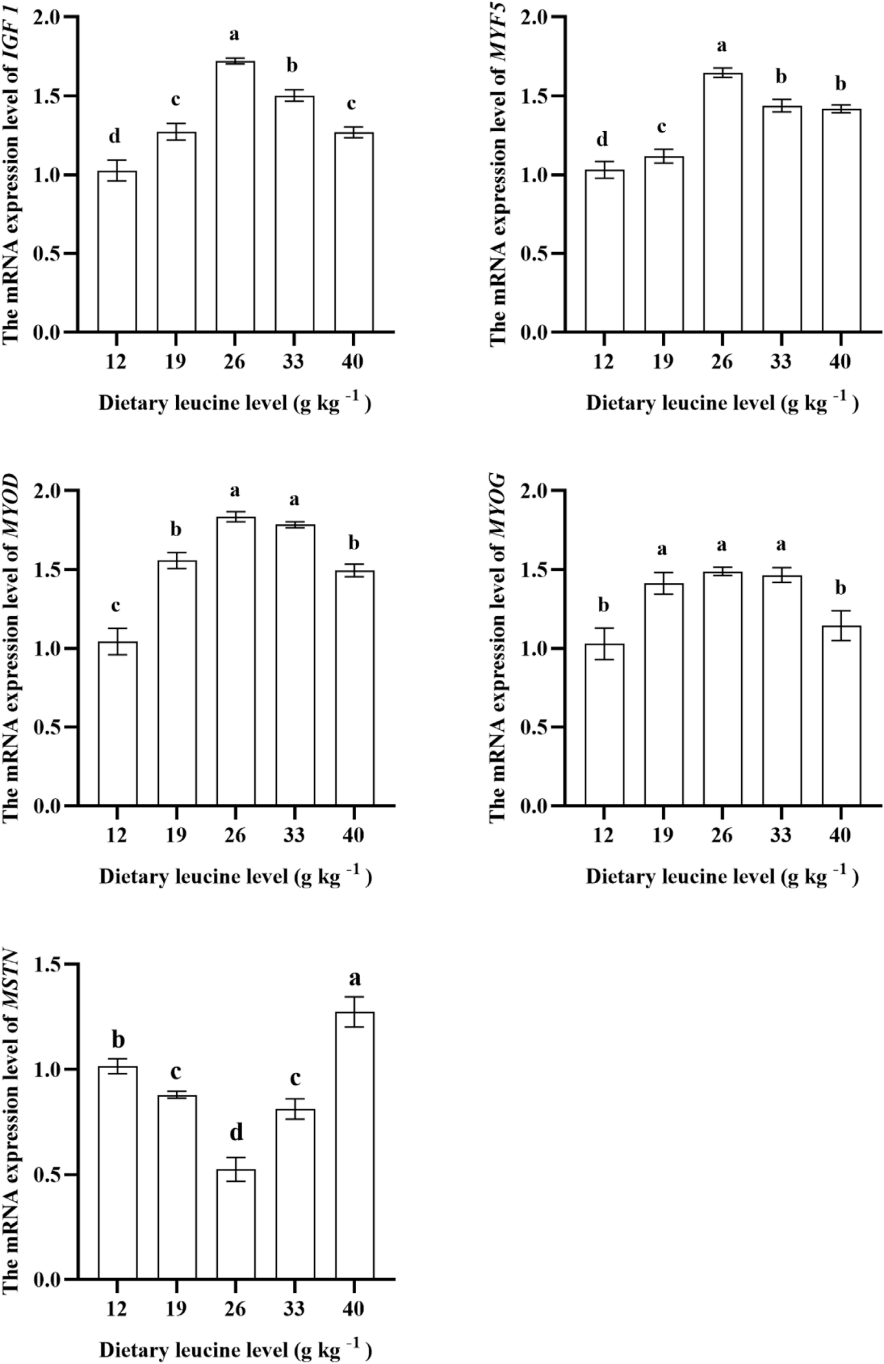
Relative expression of *IGF 1, MYF5, MYOD, MYOG*, and *MSTN* genes in the muscle of juvenile yellow catfish fed diets supplemented with various levels of leucine for 56 days. Values are expressed as the mean ± SE (*n* = 3). The same lowercase letters are not significantly different as determined by Duncan’s test between different treatment means, and error bars indicate the standard error (*p* < 0.05).

As shown in Figure 4, the expression levels of *IGF 1* and *MYF 5* genes in the muscle were significantly upregulated with increase in dietary leucine levels up to 26.00 g kg^−1^ (*p* < 0.05), but those values significantly downregulated with a further increase in dietary leucine (*p* < 0.05). The highest *MYOD* expression was found in 26.00 and 33.00 g kg^−1^ groups (*p* < 0.05). The *MYOG* expression in 19.00, 26.00, and 33.00 g kg^−1^ groups was higher than that in 12.00 and 40 g kg^−1^ groups (*p* < 0.05). The *MSTN* expression in the 26.00 g kg^−1^ group was lower than that in other groups (*p* < 0.05).

### 3.5 Liver proinflammatory gene expression

**FIGURE 5:**
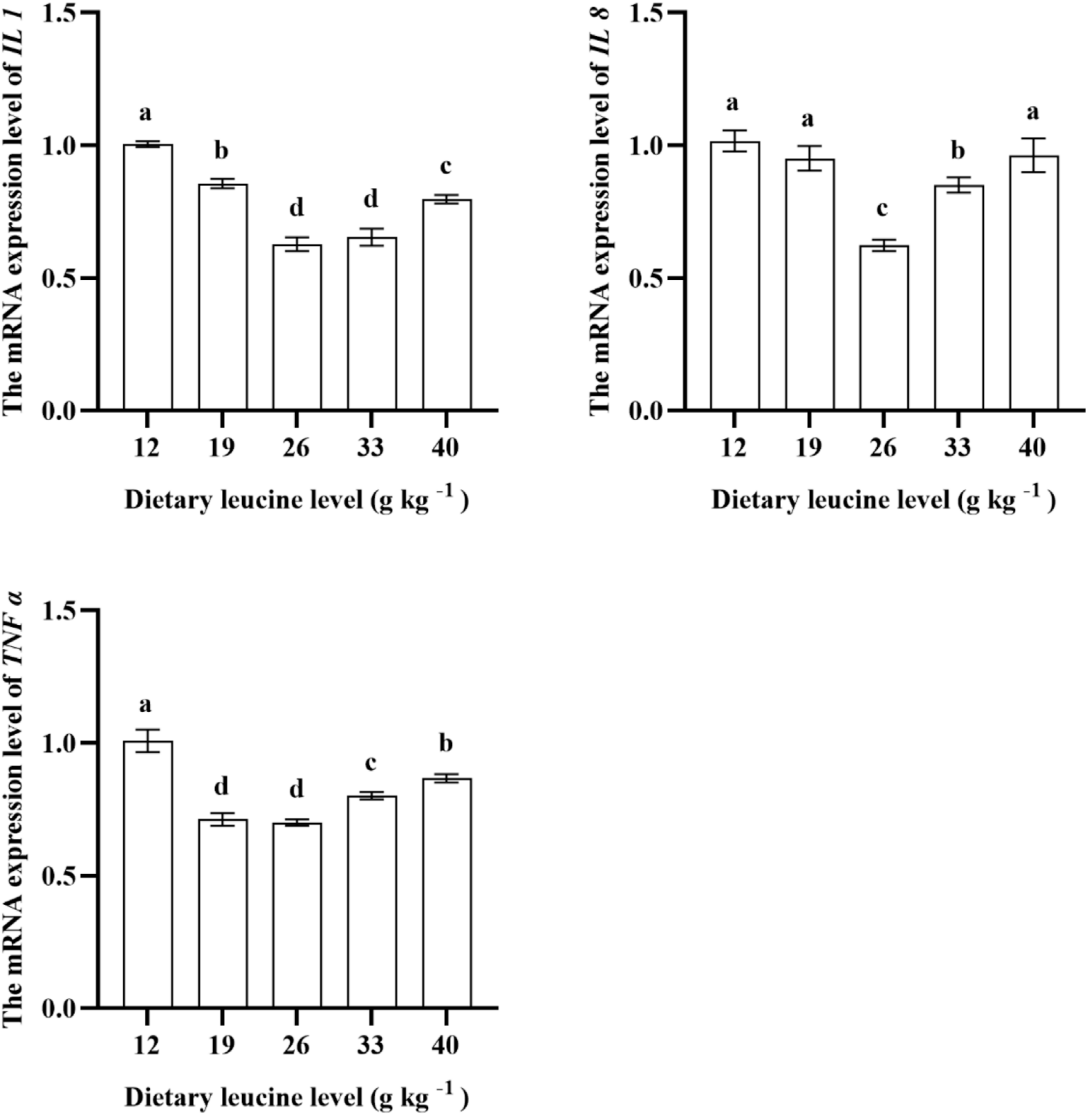
Relative expression of *IL 1*, *IL 8*, and *TNF α* genes in the liver of juvenile yellow catfish fed diets supplemented with various levels of leucine for 56 days. Values are expressed as the mean ± SE (*n* = 3). The same lowercase letters are not significantly different as determined by Duncan’s test between different treatment means, and error bars indicate the standard error (*p* < 0.05).

As shown in Figure 5, the lowest liver *IL 1* gene expression was found in 26.00 and 33.00 g kg^−1^ groups (*p* < 0.05). The *IL 8* expression in the muscle was significantly downregulated with increase in dietary leucine levels up to 26.00 g kg^−1^ (*p* < 0.05), but that value significantly upregulated with a further increase in dietary leucine (*p* < 0.05). The *TNFɑ* expression levels in 19.00 and 26.00 g kg^−1^ groups were the lowest (*p* < 0.05).

### 3.6 Correlation analysis

**FIGURE 6:**
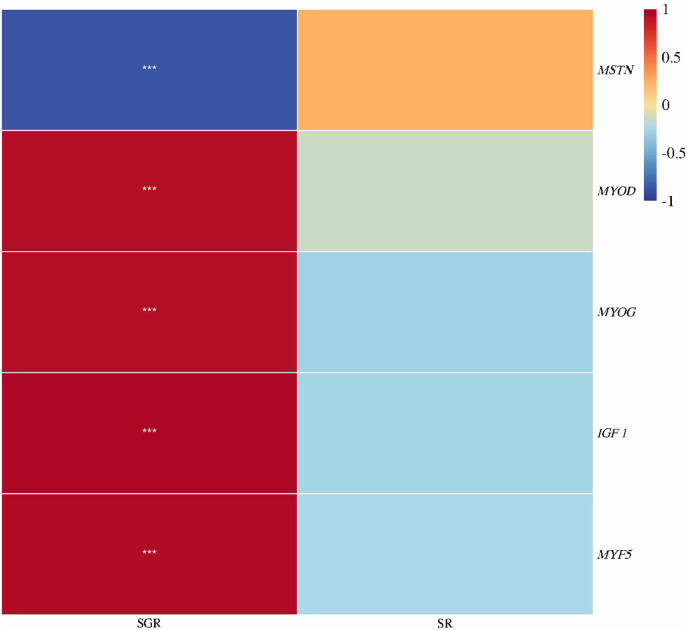
Correlation heatmap analysis between the specific growth rate (SGR) or survival rate (SR) and muscle growth genes (*IGF 1*, *MYF5*, *MYOD*, *MYOG*, and *MSTN*) of juvenile yellow catfish fed diets supplemented with various levels of leucine for 56 days (*p < 0.05; **p < 0.01; ***p < 0.001).

As shown in Figure 6, the specific growth rate was positively correlated with *IGF 1*, *MYOD*, *MYOG*, and *MYF5* expressions, but was negatively correlated with *MSTN* expression (*p* < 0.05). There was no significant correlation between survival rate and the aforementioned indexes (*p* > 0.05).

**FIGURE 7:**
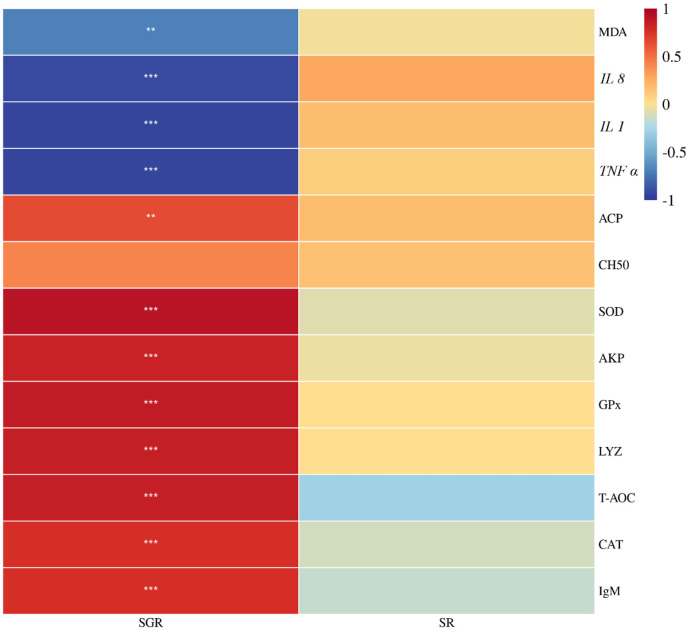
Correlation heatmap analysis between the specific growth rate (SGR) or survival rate (SR) and liver antioxidant enzyme (*T-AOC*, *SOD, CAT*, *GPx*, and *MDA*) or serum immunity (*AKP*, *ACP*, *CH50, LYZ,* and *IgM*) or proinflammatory (*IL 1, IL 8,* and *TNF α*) of juvenile yellow catfish fed diets supplemented with various levels of leucine for 56 days (*p < 0.05; **p < 0.01; ***p < 0.001).

As shown in Figure 7, the specific growth rate was positively correlated with total antioxidant capacity, superoxide dismutase, catalase, glutathione peroxidase, lysozyme, acid phosphatase activities, and immunoglobulin M content, but was negatively correlated with malondialdehyde content, *IL 1*, *IL 8*, and *TNF ɑ* expressions (*p* < 0.05). There was no significant association between the survival rate and the aforementioned metrics (*p* > 0.05).

## 4 Discussion

In the present study, quadratic regression analysis of the weight gain rate, specific growth rate, and feed conversion ratio was used to evaluate the optimal dietary leucine requirement. Depending on the growth performance of each group, the optimal leucine level in *Pelteobagrus fulvidraco* diet was predicted to be 26.84–27.00 g kg^−1^ of the dry matter diet. The results of this experimental study have similar findings to those of other fish studies, such as golden pompano (27.70 g kg^−1^ of the dry diet) (Zhou et al., 2020) and largemouth bass (25.20 g kg^−1^ of the dry diet) (Yang et al., 2022), and lower than the values for hybrid grouper *Epinephelus fuscoguttatus ♀ × Epinephelus lanceolatus ♂* (32.50–34.10 g kg^−1^ of the dry diet) (Zhou et al., 2019) and grouper *Epinephelus coioides* (35.10–44.50 g kg^−1^ of the dry diet) (Niu et al., 2021), and higher than the reported values for rohu (15.00–15.70 g kg^−1^ of the dry diet) (Abidi and Khan, 2007) and olive flounder *Paralichthys olivaceus* (12.80 g kg^−1^ of the dry diet) (Kim and Lee, 2013). However, the lowest values of weight gain rate were found in 12.00 and 40.00 g kg^−1^ groups, which indicates that both of the excessively low and high leucine levels would lead to reduction growth of yellow catfish. A recent study reported that leucine deficiency in diet induced dysregulation of branched-chain amino acids, which negatively affects the activity of intestinal digestion and absorption enzymes and reduces fish growth performance (Wang et al., 2017). On the contrary, leucine as a ketogenic amino acid and excessive consumption of leucine induced the production of large amounts of ketone compounds, which are physiologically toxic and inhibit the fish growth (Yang et al., 2022).

It is worth mentioning that leucine is an activator of growth-related genes, and the two have a synergistic effect (Han et al., 2010). A recent study shows that leucine can promote the protein synthesis of largemouth bass by upregulating the related genes involved in protein synthesis, such as *IGF 1*, *S6K1*, and *TOR* (Yang et al., 2022). *IGF 1* is essential for fish growth and skeletal muscle development (Wood et al., 2005). A recent study reported that optimal dietary leucine (25.20 g kg^−1^) could stimulate muscle hypertrophy by upregulating the expression level of *IGF 1* in the muscle of largemouth bass (Yang et al., 2022). This study found that the muscle *IGF 1* expression in the 26.00 g kg^−1^ group displays the highest value. The *MPCs* were accountable for muscle development and regeneration, which were regulated by the modulator factors including *MYF5*, *MYOD*, and *MYOG*, which are positively regulated by the upstream signaling molecule *IGF 1* to control the cell proliferation of muscles (Wang et al., 2022). A prior study reported that upregulation of *MYF5*, *MYOD*, and *MYOG* expression levels can promote Atlantic halibut *Hippoglossus hippoglossus* muscle synthesis (Galloway et al., 2006). This study found the expression levels of *MYF5*, *MYOD*, and *MYOG* in 19.00, 26.00, and 33.00 g kg^−1^ groups were significantly higher than those in other groups. Interestingly, the *MSTN* gene has a negative regulatory effect on muscle proliferation (Canada et al., 2018). However, in this study, the *MSTN* expression level was significantly downregulated in the 26.00 g kg^−1^ group, which indicates that 26.00 g kg^−1^ leucine content could promote muscle growth by inhibiting *MSTN* gene expression. Combined with correlation heatmap analysis, this study found that the specific growth rate was positively correlated with *IGF 1*, *MYOD*, *MYOG*, and *MYF5* expressions, but was negatively correlated with *MSTN* expression, which demonstrates that optimal dietary leucine can effectively activate the expression level of growth-related genes.

In addition, this study also found that the whole-body protein and lipid contents reached the maximum value in the 26.00 g kg^−1^ group. The results indicate that the optimal leucine level could promote the synthesis of body proteins and lipids, which has an initiative effect on elevating muscle quality and nutritional value of yellow catfish.

The antioxidant capacity of fish is intimately related to the activity of the antioxidant enzymes (Liang et al., 2017). Superoxide dismutase, catalase, and glutathione peroxidase were important antioxidant enzymes in the biological system, which can remove the reactive oxygen species in the organism and reduce the toxic effect of reactive oxygen species and play a crucial role in the antioxidant system (Bagnyukova et al., 2003). In this study, the highest liver total antioxidant capacity, superoxide dismutase, catalase, and glutathione peroxidase activities were found in the 26.00 g kg^−1^ group, which indicates that the addition of 26.00 g kg^−1^ leucine can significantly improve the antioxidant capacity of the liver and reduce lipid peroxidation. The results of a further analogous study were reported in rohu (Giri et al., 2015). It is worth noting that the antioxidant enzyme activities in 12.00 g kg^−1^ and 40.00 g kg^−1^ groups were significantly lower than those in other groups, which indicates that both deficiency and excess of leucine could decrease the antioxidant capacity of the liver. It may be related to malondialdehyde accumulation. Malondialdehyde is considered to be a marker of lipid peroxidation (Rana et al., 2010). In this study, liver malondialdehyde content in 12.00, 19.00, and 40.00 g kg^−1^ groups was significantly elevated.

Dietary leucine level also affects the immune response of fish. This study found that the serum immune response index increased with increasing dietary leucine levels, and the highest lysozyme activity and immunoglobulin M content were found in the 26.00 g kg^−1^ leucine group. There are numerous reports of similar results in other fish species. Dietary supplementation with the 11.00–13.30 g kg^−1^ leucine can significantly improve the acid phosphatase activity in serum of grass carp (Jiang et al., 2015). Dietary supplementation with 17.20 g kg^−1^ leucine can significantly improve the 50% hemolytic complement content in the serum of blunt snout bream (Liang et al., 2017). Dietary supplementation with 27.70 g kg^−1^ leucine can significantly increase lysozyme activity in the serum of golden pompano (Zhou et al., 2020). The reason for the improved immunity may be that leucine and its methyl b-hydroxy-b-methylbutyrate (HMB) metabolite increase the number of liver-associated lymphocytes and NK cells, thus improving the immunity of animals (Nissen and Abumrad, 1997).

Amino acids are necessary for the regulation of immune response and the synthesis of cytokines (Kelly and Pearce, 2020). The previous studies reported that dietary supplementation of leucine (27.70 g kg^−1^) can downregulate the expression levels of *TNF α* and *IL 1* in golden pompano (Zhou et al., 2020), and optimal leucine content (17.20–21.40 g kg^−1^) significantly alleviated the liver proinflammatory response by reduced liver *IL 1*, *IL 8*, and *TNFα* mRNA levels in blunt snout bream (Liang et al., 2017). In this study, dietary leucine deficiency (12.00 g kg^−1^) or excess (40.00 g kg^−1^) significantly upregulated the expression levels of *IL 1*, *IL 8*, and *TNF α* in the liver compared with the 26.00 g kg^−1^ leucine group, which indicates that the optimal dietary leucine level can alleviate liver inflammatory response. Combined with correlation heatmap analysis, in this study, the specific growth rate showed a positive correlation with total antioxidant capacity, superoxide dismutase, catalase, glutathione peroxidase, lysozyme, acid phosphatase activities, and immunoglobulin M content, but was negatively correlated with malondialdehyde content, *IL 1*, *IL 8*, and *TNF ɑ* expressions. These results indicated that although dietary leucine level did not affect the survival rate of yellow catfish, malondialdehyde accumulation and inflammation caused by leucine excess or deficiency had affected the normal growth of yellow catfish.

## 5 Conclusion

In summary, optimal dietary levels of leucine is essential for the growth of yellow catfish. A dietary level of 26.00 mg kg^−1^ leucine is required for optimal growth, and according to the quadratic regression analysis, a dietary concentration of 26.84–27.00 g kg^−1^ leucine is proposed to promote the growth performance of yellow catfish.

## Data Availability

The raw data supporting the conclusion of this article will be made available by the authors, without undue reservation.
